# Neurodegenerative Proteinopathies in the Proteoform Spectrum—Tools and Challenges

**DOI:** 10.3390/ijms22031085

**Published:** 2021-01-22

**Authors:** Aneeqa Noor, Saima Zafar, Inga Zerr

**Affiliations:** 1 Department of Neurology, University Medical Center Göttingen, 37075 Göttingen, Germany; aneeqa_n@yahoo.com (A.N.); ingazerr@med.uni-goettingen.de (I.Z.); 2 German Center for Neurodegenerative Diseases (DZNE), 37075 Göttingen, Germany; 3 Biomedical Engineering and Sciences Department, School of Mechanical and Manufacturing Engineering (SMME), National University of Sciences and Technology (NUST), Bolan Road, H-12, 44000 Islamabad, Pakistan

**Keywords:** proteinopathies, prion-like proteins, proteoforms, 2D-PAGE, top-down MS, imaging MS, hydrogen/deuterium exchange mass spectrometry

## Abstract

Proteinopathy refers to a group of disorders defined by depositions of amyloids within living tissue. Neurodegenerative proteinopathies, including Alzheimer’s disease, Parkinson’s disease, Creutzfeldt–Jakob disease, and others, constitute a large fraction of these disorders. Amyloids are highly insoluble, ordered, stable, beta-sheet rich proteins. The emerging theory about the pathophysiology of neurodegenerative proteinopathies suggests that the primary amyloid-forming proteins, also known as the prion-like proteins, may exist as multiple proteoforms that contribute differentially towards the disease prognosis. It is therefore necessary to resolve these disorders on the level of proteoforms rather than the proteome. The transient and hydrophobic nature of amyloid-forming proteins and the minor post-translational alterations that lead to the formation of proteoforms require the use of highly sensitive and specialized techniques. Several conventional techniques, like gel electrophoresis and conventional mass spectrometry, have been modified to accommodate the proteoform theory and prion-like proteins. Several new ones, like imaging mass spectrometry, have also emerged. This review aims to discuss the proteoform theory of neurodegenerative disorders along with the utility of these proteomic techniques for the study of highly insoluble proteins and their associated proteoforms.

## 1. Introduction

Proteinopathies, also known as protein conformational diseases or amyloidosis, are a group of diseases associated with the deposition of misaggregated proteins in various organs [1]. These atypical protein conformations, known as amyloids, are β-sheet rich, insoluble, fibrillar assemblies that retain a uniform structure containing β-pleated sheets running perpendicular to the fiber axis [2]. With the aid of amyloid assemblies of thirty proteins, this phenomenon is reported to be the cause of almost fifty disorders [3]. Although the pathophysiology and symptoms of proteinopathies vary with the nature of misaggregated proteins and affected populations of cells, the primary cascade leading to the formation and deposition of misaggregated proteins is highly similar. Their propagation closely resembles the replication of scrapie isoform of cellular prion protein (PrPSc), the first infectious protein to be discovered in conjunction with human diseases, leading to the use of the terms ‘prion proteins’ and ‘prion-like proteins’ to describe amyloid-forming proteins [4].

**Figure 1:**
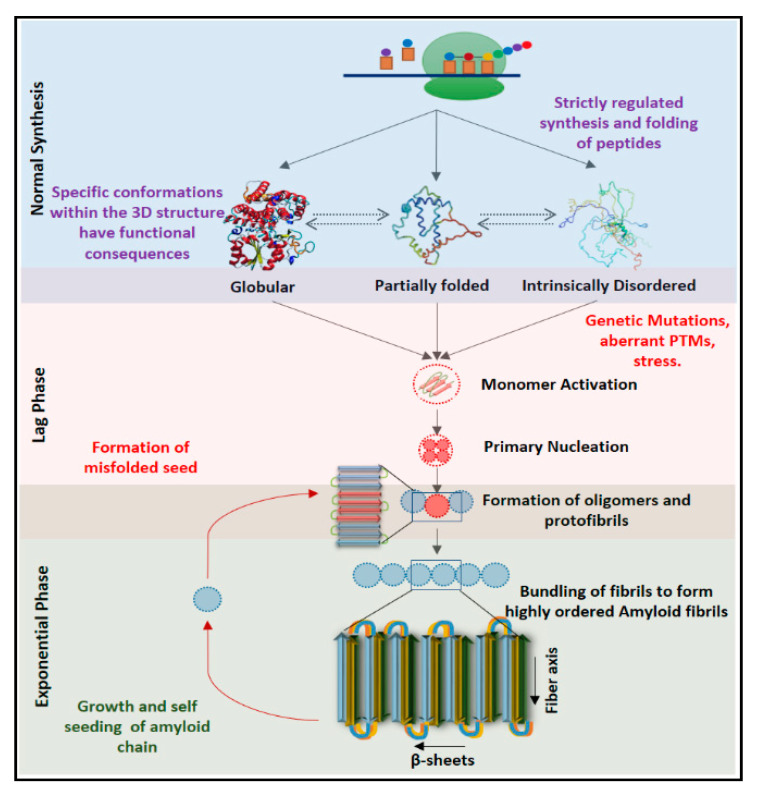
The mechanism of formation of amyloids: The organization of proteins into globular, partially folded or intrinsically disordered functional conformations is a tightly regulated process. However, mutations, aberrant cleavage or cellular stress can cause the formation of altered monomeric species. This event, known as monomer activation, destabilizes the native structure and forms the thermodynamically favorable β-sheets rich structure. The combination of these altered structures, or primary nucleation, leads to the formation of various multimers, protofibrils (2.5 to 3 nm in diameter), and fibrils (6 to 10 nm). The intertwining of protofibrils and fibrils leads to the formation of highly stable mature amyloid fibrils (60–120 nm). The primary event of nucleation and fibril formation is relatively slow and is referred to as the lag phase of growth. As more native proteins mimic the structure of misfolded seeds, the growth of amyloid fibrils reaches an exponential phase leading to rapid accumulation of aggregates [7,8].

**Figure 2:**
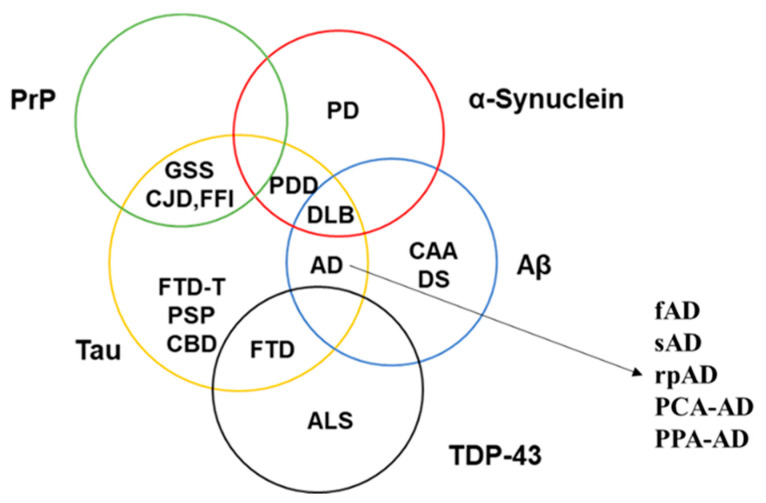
Involvement of known prion-like proteins in multiple neurodegenerative disorders. The figure depicts the overlapping pathological profile of PrP (green circle), α-Synuclein (red circle), Aβ (blue circle), Tau (yellow circle), and TDP-43 (black circle). Each of the stated disorders have further clinical variants (as shown in the case of AD), thereby complicating the role of prion-like proteins in bringing about the observed pathology. PDD—Parkinson’s disease with dementia; DS—Down’s syndrome; FTD-T—frontotemporal dementia with tau pathology; fAD—familial AD; sAD—sporadic AD; rpAD—rapidly-progressive AD; PCA-AD—posterior cortical atrophy–AD; PPA-AD—primary progressive aphasia with AD.

The conversion of physiological proteins to pathological amyloid fibrils is an intriguing, albeit partially understood, process initiated by molecular stressors that collapse the native structure by breaking the backbone of the helix and prompting interactions between side chains of resident amino acids [5]. The peptide then refolds into a compact β-sheet rich secondary structure that is stabilized by the presence of electrostatic interactions. The conversion of native helical structure to a thermodynamically favorable β-sheet rich conformation is also known as ‘monomer activation’. These misfolded units can self-replicate by interacting with their physiological counterparts and altering their conformation via deformed templating [6]. The combination of these altered structures, or primary nucleation, leads to the formation of an aggregate that can seed the formation of amyloid fibrils [7]. These seeds undergo a repetitive cycle that involves the assembly of multiple toxic oligomeric species leading to the formation of various multimers, protofibrils (2.5 to 3 nm in diameter) and fibrils (a combination of two strands with a diameter of 6 to 10 nm; [8]). The primary event of nucleation and fibril formation is relatively slow and is referred to as the lag phase of growth. The intertwining of protofibrils and fibrils leads to the formation of mature fibrils that are 60–120 nm in diameter (Figure 1; [2]). X-ray diffraction and nuclear magnetic resonance analysis shows a spacing of approximately 10 Å between the layers of beta-sheets and approximately 4.7 Å between multiple β-strands, depicting a uniform and stable assembly [7]. The addition of monomers to fibrils changes their conformation so that it matches the residues present in the aggregates, leading to the growth of amyloid fibrils, a step referred to as ‘secondary nucleation’ [9]. At this point, the growth of amyloid fibrils reaches an exponential phase causing rapid accumulation of aggregates (Figure 1). Exosome-mediated transport can then spread the oligomeric species to other parts of the affected organ, spreading the pathology [10,11]. Depending on the post-translational processing and three-dimensional folding, each prion-like protein can generate diverse variants (proteoforms) that may contribute differentially towards disease prognosis.

Owing to the distinct, possibly optimal, redox and biochemical profile of the nervous tissue, a major fraction of proteinopathies in humans are associated with the central nervous system, making them a key problem for neuroscientists. In most cases, minuscule variations in protein sequences can lead to structural variations in their three-dimensional conformations, generate amyloids, and alter their seeding and infectious capabilities and the phenotype of associated disease [12,13]. The key to solving the riddle of these proteinopathies lies in a thorough investigation of their proteinaceous culprits. However, a lack of appropriate techniques to purify, identify, and characterize remains a major hurdle despite a collective effort of several research groups around the globe.

Strides in expression, structural and functional proteomics, to accommodate highly fibrillar and polymorphic prion-like proteins, are the need of the hour. The current review aims to highlight variants of amyloidogenic proteins involved in proteinopathies and describe the utility and limitations of proteomic tools in their study.

## 2. Neurodegenerative Proteinopathies

Some of the most common neurodegenerative disorders—Alzheimer’s disease (AD), Parkinson’s disease (PD), Creutzfeldt–Jacob disease (CJD), Dementia with Lewy bodies (DLB), Huntington disease (HD), and Amyloid Lateral Sclerosis (ALS) —are proteinopathies. Together, these diseases affect millions of lives around the world and have devastating economic implications. AD, the most frequently diagnosed among these listed diseases, affects almost one-tenth of the population above 65 years of age [14]. The number of people suffering from these diseases is increasing rapidly with an increase in life expectancy and it is predicted that by 2050, 135.46 million people will be living with various types of neurodegenerative dementias [15]. Despite the attention of the scientific community, these disorders are far from resolved. The patients can be treated to alleviate the symptoms, but the lack of a cure still means that such a diagnosis can seal their fate.

Although proteinopathies present similarities in their pathological mechanisms, the psychological and physiological symptoms of all these disorders vary and depend on the region of the brain affected. A summary of age at onset, primary sites of pathology, and common symptoms of major neurodegenerative proteinopathies is presented in Table 1. These variations, in turn, are dictated by the proteins that are involved in amyloid formation (Table 2).

In addition to similarities in the mechanism of propagation, prion-like proteins have also adapted another interesting aspect of PrPSc biology. PrPSc can give rise to several clinical variants of prion diseases. This heterogeneity has been attributed to the existence of distinct PrP strains. Strains are defined as conformers of a specific amyloidogenic protein, in this case PrPSc, that differ with respect to their transmission, brain-lesion profiles, incubation periods, and disease phenotypes along with certain biochemical characteristics like Post-translational modifications, sensitivity to proteinase-K, and electrophoretic mobility. The distinct conformational characteristics of each PrP strain are transmitted into the host where it propagates and causes distinct phenotypes [40]. Codon 129 polymorphism gives rise to at least three known strains of PrP in humans [41].

Strain theory is now applicable to most prion-like proteins (Figure 2) [42,43]. α-Synuclein, for example, has been known to be the culprit behind characteristically distinct pathologies, i.e., PD, DLB, and multiple system atrophy, while microtubule-associated protein tau is involved in multiple different tauopathies either as the primary cause or as a co-pathology [44,45]. In the case of Aβ, it has been known for several years that different proteoforms vary in their capability to form amyloids, seeding proficiencies, three-dimensional conformations, transport mechanisms and toxicities [46,47]. Each proteoform can adopt and propagate in multiple conformations [48]. These conformers do not only possess distinct biochemical signatures but also have different stabilities, distribution and morphology in the brain [49]. Moreover, accumulating evidence shows that many neurodegenerative proteinopathies can exist as rapidly progressive and other clinically distinct variants even though the underlying prion-like proteins and mechanisms are the same [50,51]. The capability of one protein to give rise to clinically distinct disorders and alter the progression of a disease has further complicated the characterization of neurodegenerative proteinopathies.

The study of prion-like proteins now encompasses the study of all variants/proteoforms rather than focusing on one parent entity. The existence of proteins as different functional variants is a known fact. These functional variants dictate the localization, uptake, recycling, and biological functions of a protein. In the case of prion-like proteins, the presence of distinct variants involved in neurodegenerative proteinopathies has been verified by several groups over the past two decades [26,28,52]. Although several different terms have been previously used in the literature to classify these variations, any prion-like protein can have:

1. Genetic variants (based on mutations).
2. Isoforms (based on differences in post-transcriptional modifications).
3. Proteoforms (based on differences in post-translation processing and three-dimensional conformation).
4. Strains (based on differences in infectivity and incubation periods).

With the acceptance of the notion that different isoforms, proteoforms, or strains of prion-like proteins may differ with respect to their molecular insult mechanisms and dictate the prognosis of associated pathology, the availability of high-resolution data about the sequence and structure has become the key in characterizing, diagnosing, and treating neurodegenerative proteinopathies [53,54,55,56,57,58]. It is therefore mandatory to establish tools that can provide insight into minor changes within the sequence, post-translational processing, and structure of a protein in its undigested form or native conformations.

## 3. Utilizing Proteomic Platforms to Understand Neurodegenerative Proteinopathies

The identification and characterization of proteoforms and strains require high-resolution proteomic techniques that can analyze proteins in their intact native state. As the focus of the scientific community is shifting now from proteome to proteoforms, several such techniques are being refined and developed. Two-dimensional gel electrophoresis (2D-GE) is becoming popular again as an important method to visualize the presence of proteoforms of the target protein [59]. Based on the preliminary evidence from gel-based techniques, the proteoforms can then be sequenced directly in their native forms using top-down mass spectrometry [60,61]. The recently developed imaging mass spectrometry (IMS) can localize proteoforms in the tissues directly, diminishing the need for immunofluorescence microscopy and proteoform-specific sensitive antibodies [62,63]. In addition, conformational variation between related proteoforms can be understood by utilizing hydrogen/deuterium exchange mass spectrometry [64].

Theoretically, these techniques should identify and characterize any protein and its associated proteoforms isolated from a range of biological specimens. However, the attributes of prion-like proteins make them especially challenging. Biochemically, these proteins are highly insoluble, fibrillar, heterogenous, and transient in nature. Therefore, minor changes in detergents, chaotropes, solvents, pH, plasticware, and storage conditions can result in the formation of multimers and fibrils and/or loss of proteins. The results require careful analysis to rule out any technical artifacts. In some cases, like for AD-associated Aβ peptides, their quantity is very low, therefore requiring a large amount of brain samples or enrichment techniques. However, careful planning and execution of experiments can provide useful insights into the molecular mechanisms involved in neurodegenerative proteinopathies.

In the following sections the application, and challenges, of proteomic techniques to expand our knowledge from the proteome of neurodegenerative proteinopathies to the proteoforms of associated prion-like proteins will be discussed.

### 3.1. Two-Dimensional Gel Electrophoresis (2D-GE)

Gel electrophoresis, coupled with immunoblotting and mass spectrometry, is an integral tool for proteomic labs and provides valuable insights into the physiological and pathological functions of proteins [65]. Even the most straight-forward gel electrophoresis experiments allow the visualization of the proteome and quantification of various proteins in comparison to others. The basic principle of this technique relies on the efficient separation of proteins based on their biochemical characteristics. One-dimensional gel electrophoresis performs the separation of proteins based on their respective molecular weight [66]. On the other hand, its two-dimensional counterpart utilizes the differences in isoelectric points in addition to molecular weights to separate proteins enabling the visualization of thousands of proteins [67]. The latter tool is ideal for separating the intact proteoforms of any protein even if they have minor differences in biochemical properties in response to differential cleavage and post-translational modifications [68]. This attribute of 2D-GE has increased its popularity over the last few years and its applicability towards the study of proteoforms of prion-like proteins is becoming increasingly evident [69].

The analysis of proteins through gel-based techniques requires the proteins to be in a soluble, denatured, and monomeric state as well as the retention of epitopes essential for the immunoblotting or pull-down assays. In addition, for 2D-GE, the proteins should retain their native charges. Bringing fibrillar assemblies of prion-like proteins into this state requires an understanding about the type of aggregates, their constituent proteins and their amount in the tissue [70]. Prion-like proteins are not soluble in most commonly used detergents and chaotropes. In fact, some of these agents, including Triton X-100, Tween-20, CHAPS, and sodium dodecyl sulphate (low concentrations), enhance the insolubility in certain cases and increase the formation of multimers in the process. Furthermore, the solubility of different conformations, i.e., monomers, multimers, and plaques, of the same target protein is different, thus requiring a sequential fractionation-based approach to isolate all aggregates from a sample [71]. Therefore, the cocktail of detergents, chaotropic agents, and reducing agents, and the pH of the system must be tailored according to the target protein and its conformation. If improperly solubilized, they fail to enter the gel, compromising the analysis [72]. Proteins become highly insoluble as they reach their isoelectric points (pI) and therefore their ability to form aggregates increases [73]. The selected rehydration buffer for 2D-GE must also ensure that the proteins stay soluble throughout the running period.

Mild agents, like phosphate buffered saline (PBS), have been used for the isolation of smaller assemblies, i.e., monomers and oligomers [71]. Larger fibrillar assemblies need stronger ionic detergents, mostly sodium dodecyl sulphate (SDS) or sodium N-lauroylsarcosinate (Sarkosyl), for effective solubilization [72]. However, the use of ionic detergents disturbs the native charges that are required for 2D-GE and interferes with mass spectrometric analysis [74,75]. High concentrations of chaotropic agents, including urea and guanidine hydrochloride, are also very effective for the solubilization of fibrillar assemblies [76,77,78]. Amongst the chaotropes, uncharged urea is most commonly used for the extraction of proteins for subsequent 2D-GE. Its combination with low concentrations of CHAPS, a zwitterionic detergent, preserves the native charges of proteins and prevents the formation of aggregates [79]. In addition, formic acid, trifluoracetic acid and other organic acids can also be employed for the solubilization of fibrillar assemblies [70]. These solvents can be exchanged or neutralized prior to analysis.

**Figure 3:**
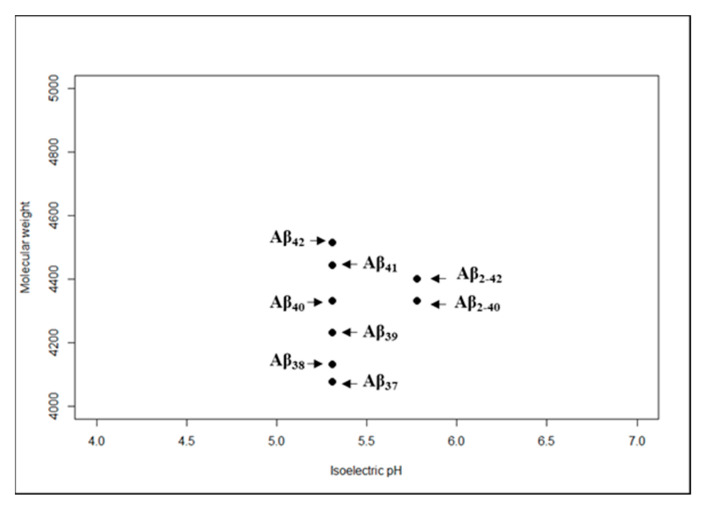
Virtual 2D map for AD-associated prion-like proteins. Differences in electrophoretic mobility of Aβ proteoforms based on variations in post-translational cleavage.

Once the proteins are solubilized with their native charges intact, they can be subjected to isoelectric focusing using a range of commercially available IPG strips. The separation on the second dimension using polyacrylamide gels requires additional considerations. The presence of SDS in polyacrylamide gel electrophoresis has drastic effects on the mobility of some prion-like proteins. In the case of Aβ, this phenomenon has been reported previously [80]. Supplementation of polyacrylamide gels with urea nullifies the effects of SDS and improves the resolution of proteoforms even if the differences among them are based on single amino acid only [79,81]. The gels can then be subjected to immunoblotting. Using 2D-GE, in comparison to 1D-GE, would not require specific antibodies against all targeted proteoforms since they are already resolved into specific spots instead of a single band. One antibody against the parent proteoform would detect all its associated proteoforms. The virtual 2D-GE based immunoblots for Aβ are shown in Figure 3.

The optimization of 2D-GE for prion-like proteins has offered a straightforward and economical preliminary approach to detect the presence of proteoforms. It can be employed to obtain information about glycosylation, sialylation, presence/absence of GPI-anchor, and differences in post-PK cleavage pattern for PrPSc from brains and cerebrospinal fluid of patients [82,83,84]. Similarly, in addition to splice-variants, phosphorylation, acetylation, O-GlcNAcylation of tau proteins have been studied using 2D-GE [85,86,87]. Combined with mass spectrometry, 2D-GE is a powerful tool for the identification and the analysis of known and novel prion-like proteins.

### 3.2. ESI and MALDI Based Top-Down Mass Spectrometry

Top-down proteomics is a relatively new addition to the field of mass spectrometry. Although still in the development stages, this technique is ideal for the study of proteoforms. Unlike bottom-up proteomics, it does not involve the digestion of proteins to peptides before the analysis and thus no information about the sequence and its post-translational modifications is lost. The proteins can be confidently annotated as the complete sequence is available. The analysis, however, is much more complicated than bottom-up proteomics as the digested peptides are not only easier to solubilize and fragment, they also have ample bioinformatic support. In addition, the number of total proteoforms is far greater than the total number of functional gene products initially expected to exist. Hence top-down proteomics requires more sensitive and high-resolution mass spectrometric techniques [88]. This technique has been used for the study of prokaryotic proteoforms for over two decades now and is rapidly advancing to accommodate the complicated world of eukaryotic proteoforms [89,90].

Prior to analysis using this technique, proteins need to be extracted and purified in solvents compatible with mass spectrometry (extraction and purification discussed in the previous section). The introduction of complex mixtures into columns and analyzers is performed by combining two fractionation techniques or two-dimensional fractionation. The differential size, isoelectric pH, molecular weight, or solubility of proteins can be targeted. Reverse-phase liquid chromatography can be coupled with size exclusion chromatography, hydrophobic interaction chromatography, capillary isoelectric focusing, or capillary zone gel electrophoresis to achieve this goal. The intact proteins are converted into intact ions using soft ionization methods like electrospray ionization (ESI) and matrix-assisted laser desorption ionization (MALDI). They are then fragmented, and the MS/MS data are utilized for identification. Mostly ESI coupled with Fourier-transform ion cyclotron resonance (FT-ICR) analyzer is utilized for the analysis of intact proteins because of their ability to generate multiple charged fragments and high-resolution capabilities, respectively. Known sequences from parent proteoforms can be used to comprehend the differences within associated proteoforms [91].

An additional advantage of top-down mass spectrometry is the quantification of native proteoforms. As the measurements involve complete sequence coverage of intact analytes, results from top-down mass spectrometry can be directly used to quantify proteoforms [92]. This approach, after refinement, can be potentially used as a specific and sensitive substitute to enzyme-linked immunosorbent assays. Moreover, the top-down approach in combination with hydrogen/deuterium exchange mass spectrometry, discussed in Section 3.4, can be employed to study conformational variations among associated proteoforms in their native form thereby providing valuable insights into functionally-relevant structural differences [93].

Top-down proteomics is becoming increasingly applicable for the study of eukaryotic proteome and proteoforms [92,94]. It is also being used for the study of native prion-like proteins. α-Synuclein has been subjected to this technique to identify its metal-binding domains that contribute towards the pathology of Parkinson’s disease [95]. Similarly, the native structure of tau and its interactions with aggregation inhibitors have also been targeted [96]. The heterogeneity of Aβ proteoforms in AD has also been established using a top-down approach [26]. The number of publications in this field is increasing every year and soon, it may become the primary tool for the study of proteinopathies.

### 3.3. MALDI Imaging

The spatial distribution of proteins in tissues, in addition to their identification and expression, is necessary for its complete characterization in physiological and pathological conditions. For several decades, immunostaining and immunofluorescence have fulfilled this aim [97]. However, they require specific and sensitive antibodies for each target. In the case of proteoforms, this condition cannot always be met as the proteoforms of the same proteins can vary because of slightly altered post-translational modifications and the antibodies might not be specific enough to detect these alterations. Secondly, antibody-based methods require prior knowledge about the target, and the discovery of novel proteins is not possible.

The alternative to these conventional techniques is MALDI imaging mass spectrometry (MALDI-IMS). It involves the MS-based detection of analytes on frozen or paraffin-embedded tissue samples directly. It was first reported in the 1990s and is becoming popular for its ability to profile proteins, lipids, metabolites, glycoproteins, and nucleotides in academic and clinical settings [62,98]. It can be used for the analysis of proteins through both bottom-up and top-down approaches. The protocols, equipment, and software for MALDI-IMS are advancing rapidly and have already been developed to accommodate the analysis of proteoforms and prion-like proteins [99,100,101].

The preparation of samples for MALDI-IMS is relatively straightforward. It involves sectioning of tissues and their mounting on indium-tin oxide coated slides. Ethanol fixation is employed to limit the delocalization of proteins. Impurities, like salts and lipids, are removed using a combination of organic solvents, and the tissue is coated with trypsin (if digestion of the analyte is required). The matrix is selected based on the size and biochemical attributes of the target analyte and uniformly coated on the tissue using automated sprayers followed by analysis. Simultaneously, the same tissue can be stained using cresyl violet, hematoxylin and eosin, methylene blue, or immunohistochemistry to visualize the cellular population. These stained images can be overlaid with MALDI-IMS images to assign each protein to a certain cellular population or region of interest. There are several reviews and methodology papers available for fine-tuning the protocols according to the requirements of each protein [100,102].

The samples can be analyzed using time of flight (TOF) or the more sensitive and accurate FT-ICR analyzers [100]. The m/z values from the detected peaks can be compared against protein databases or, in the case of novel proteoforms, in silico fragmentation results to annotate proteins. The results can be validated by analysis of proteins from the target region or whole tissue extract using Liquid chromatography-electrospray ionization-Tandem mass spectrometry LC-ESI-MS/MS to obtain sequences of target peptides. As the MALDI-based approach results in the generation of singly charged species, the targeted analysis of the m/z ratio is less complicated, especially in the top-down approach for smaller proteins and can be utilized to understand differences among associated proteoforms. However, the analysis of larger proteins in their native states is still challenging and requires the integration of various related mass spectrometry-based methods to detect and confidently annotate proteins [103].

**Figure 4:**
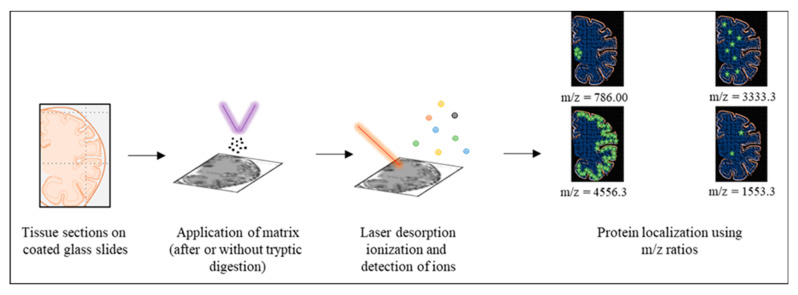
Summarized methodology matrix-assisted laser desorption ionization-imaging mass spectrometry (MALDI-IMS) for Aβ proteoforms. A section of the tissue is deposited onto conductive slides (step 1) and coated uniformly with the appropriate matrix solution (step 2). Matrix and sample are desorbed and ionized upon the application of ultraviolet laser (red line; step 3). These gaseous ions (colored dots) are then analyzed. Targeted proteins can be localized by selecting the m/z value corresponding to their ions as shown for the m/z ratio of 786, 3333.3, 4556.3 and 1553.3 with green stars in the figure. The relative number of stars depicts the hypothetical signal intensity for the selected m/z ratio.

In the context of the neurodegenerative proteinopathies, this technique has been especially useful for the AD-associated Aβ peptide and has been employed to identify the signature of Aβ proteoforms and distribution in AD (Figure 4) [104,105]. Aβ is a small peptide (4kDa) and is conveniently detected directly on the tissue if the quality of tissue is good and impurities have been removed effectively. Other prion-like proteins, including tau, have also been studied using MALDI-IMS [106]. It has also been utilized to unveil aberrations in brain architecture caused by prion-like pathologies [107].

In addition to providing useful insights into the pathological roles of prion-like proteins and their proteoforms, this technique can also be used to detect the localization of pharmacologically active substances and their effects on pathological proteoforms [98]. Although still under active research, this attribute of MALDI-IMS will be useful for understanding and treating neurodegenerative proteinopathies.

### 3.4. Hydrogen/Deuterium Exchange Mass Spectrometry

The alterations in sequence and post-translational modifications of proteins mainly impart functional changes by modulating the three-dimensional structure of proteins. Structural alterations in fibrillar aggregates formed by variants of prion-like proteins are an important source of variability in their toxicity and are frequently used as an explanation of heterogeneity in the clinical presentation of proteinopathies. Slight alterations in structure translate to modified biochemical properties, incubation periods, propagation, and toxicities. Common structural techniques, including electron microscopy and atomic force microscopy, have been popular for obtaining low-resolution images of amyloid morphology whereas X-ray fiber diffraction, nuclear magnetic resonance and Fourier-transform infrared spectroscopy have been used to provide detailed information about secondary structures and 3D organization [108]. However, the prerequisite of purified and highly concentrated amyloids in most of these techniques is a major drawback. With the advances in hydrogen/deuterium exchange mass spectrometry, proteomics has stepped into the field of structural biology and is providing critical knowledge about prion-like proteins.

In contrast to low resolution imaging and infrared spectroscopy techniques, hydrogen/deuterium exchange mass spectrometry (HDX-MS) is based on the ease of isotopic exchange on the peptide chain. The rate of this exchange is dependent on the 3D folded state of the protein, the chemical properties of amino acid residues, their intrinsic bonding, and the aggregation status, and can therefore depict the physiological states effectively [109]. This technique is becoming increasingly useful for assessing the conformational diversity of prions and associating them with the clinical heterogeneity of neurodegenerative disorders [110,111,112,113].

## 4. Conclusions

The heterogeneity of neurodegenerative proteinopathies has rapidly expanded the study of this group of diseases from the proteome to proteoforms and the availability of high-throughput and sensitive proteomic techniques has become the need of the hour. Although some conventional techniques, like 2D-GE, are already usable for this purpose, several top-down and structural mass spectrometric techniques are being optimized to enhance our knowledge of this field (Table 3). The utility of these tools for the analysis of prion-like proteins associated with neurodegenerative proteinopathies will dictate the advancements in understanding, diagnosing, and treating these disorders.

## Figures and Tables

**Table 1:** Age at onset, affected brain regions, and common symptoms of major neurodegenerative proteinopathies. Age at onset represents a range, rather than mean, due to multiple clinical variants of each of these disorders. * sporadic CJD.

Proteinopathy	Age at Onset (Years)	Primary Region	Common Symptoms
AD	40–65 (early and late-onset variants)	Hippocampus and entorhinal cortex.	Memory and language impairment and visuospatial deficits. [16,17]
PD	40–50	Substantia nigra (midbrain).	Rigidity, resting tremor and bradykinesia. [18]
sCJD *	44–70 (depends on subtype)	Cerebral cortex and cerebellum.	Cognitive impairment and myoclonus. [19]
DLB	50–80	Midbrain and neocortex.	Parkinsonian syndrome, autonomic and sleep fluctuations and hallucinations. [20]
HD	20–49	Caudate nucleus and putamen (basal ganglia).	Choreiform movements, emotional and behavioral alterations, bradykinesia. [21]
ALS	45–55	Motor neurons.	Focal muscle wasting, spasticity and flexor spasms. [22,23]

**Table 2:** A summary of the structure and variants of major amyloidogenic proteins associated with neurodegenerative proteinopathies.

Amyloids	Precursor Protein	Associated Diseases	Proteoforms or Other Variants
Aβ	Amyloid beta A4 protein: Intrinsically disordered protein with 770 residues	AD, Cerebral amyloid angiopathy (CAA) [24,25].	26 differentially truncated and post translationally modified proteoforms [26]
α-Synuclein	Alpha Synuclein: Intrinsically disordered protein with 140 residues	PD and DLB [27]	11 differentially truncated and post translationally modified proteoforms [28]
PrPSc	Major prion protein: Intrinsically disordered protein with 253 amino acids	CJD, Fatal Familial Insomnia (FFI), Gerstmann-Straussler-Scheinker disease (GSS), Huntington disease-like type 1 (HDL1), Kuru and Spongiform encephalopathy [29]	2 Proteoforms based on Proteinase-K resistance Genetic variants (codon 129 polymorphism). [30]
ASOD	Superoxide dismutase: Intrinsically disordered protein with 154 amino acids	ALS—TDP-43 amyloids also involved. [31,32]	Genetic variants. No proteoforms reported yet. [33]
ATau	Microtubule-associated protein tau: Intrinsically disordered protein with 758 amino acids	Frontotemporal dementia (FTD), AD, Progressive Supranuclear Palsy (PSP), Corticobasal degeneration (CBD), Pick’s disease, Argyrophilic grain disease, Dementia with Lewy bodies and Parkinsonism linked to chromosome 17. [34]	Six isoforms. Differentially post translationally modified proteoforms. [35]
ATTR	Transthyretin: Mostly β-sheet with 147 amino acids	Familial Amyloid polyneuropathy, Leptomeningeal amyloidosis. [36]	Differentially oxidized proteoforms. [37]
AHtt	Huntington: Intrinsically disordered protein with 3142 residues	Huntington disease. [38]	Differentially post translationally modified proteoforms. [39]

**Table 3:** A summary of the capabilities and considerations for the proteomic tools discussed in this review. The previously studied amyloids, using each of these techniques, have also been stated.

Technique	Utility for Amyloids	Samples	Previously Targetted Amyloids
2D-GE	Resolves minor biochemical variations among proteoforms by targeting isoelectric points and molecular weights.	Solubilized proteins with native charges preferably monomeric species (multimeric species may provide misleading results if more than one proteoform is involved). Buffers selected must not induce aggregation under experimental conditions.	PrP, Aβ, Tau
Top-Down MS	Identifies proteoforms and their post-translational modifications in their native forms.	Undigested proteins in their native conformations. Buffers that prevent aggregation but do not affect the spectrum of target. In case of MALDI, matices have to be tested for their capability to ionize the target.	Aβ, tau, α-Synuclein
MALDI IMS	Locates proteins via in situ identification of proteoforms.	Paraffin-embedded or frozen tissue sections. Matices have to be tested for their capability to ionize the target.	Aβ, tau
HDX-MS	Depicts 3D structures of proteins.	Undenatured, undigested proteins in their native conformations. Experimental conditions have to be carefully controlled to prevent uneven deutrium labelling among replicates.	PrP, Aβ

## Data Availability

Not applicable.
